# Case Report: Improvement of functional dyspepsia using eight constitution acupuncture and eight constitution diet – A report of three cases

**DOI:** 10.3389/fmed.2025.1545687

**Published:** 2025-07-23

**Authors:** Nahyun Cho, Younkuk Choi, Heekyung Kim, Heeyoung Moon, Younbyoung Chae, Sungha Kim, Jungtae Leem

**Affiliations:** ^1^Department of Diagnostics, College of Korean Medicine, Wonkwang University, Iksan-si, Jeonbuk-do, Republic of Korea; ^2^East-West Cancer Center, Cheonan Korean Medical Hospital, Daejeon University, Cheonan-si, Chungcheongnam-do, Republic of Korea; ^3^Department of Clinical Research Design and Evaluation, Samsung Advanced Institute for Health Sciences and Technology, Sungkyunkwan University, Seoul, Republic of Korea; ^4^Gangnam-Shingwang ECM Clinic, Seoul, Republic of Korea; ^5^Yebon ECM Clinic, Seoul, Republic of Korea; ^6^Department of Meridian and Acupoints, College of Korean Medicine, Semyung University, Jecheon, Republic of Korea; ^7^Acupuncture and Meridian Science Research Center, Kyung Hee University, Seoul, Republic of Korea; ^8^KM Science Research Division, Korea Institute of Oriental Medicine, Daejeon, Republic of Korea; ^9^Research Center of Traditional Korean Medicine, College of Korean Medicine, Wonkwang University, Iksan-si, Jeonbuk-do, Republic of Korea; ^10^Department of Il-won Integrated Medicine, Wonkwang University Korean Medicine Hospital, Iksan-si, Jeollabuk-do, Republic of Korea

**Keywords:** dyspepsia, acupuncture therapy, diet therapy, personalized medicine, case report

## Abstract

**Background:**

Functional dyspepsia (FD) is a common gastrointestinal disorder affecting 10–20% of the global population. This case series aimed to report the clinical outcomes of three patients with FD who were treated with eight constitution acupuncture (ECA) and eight constitution diet (ECD), a personalized treatment approach based on the eight constitution medicine (ECM) theory.

**Methods:**

Three patients with chronic FD were retrospectively selected from two Korean medical clinics. Each patient underwent ECA and ECD according to their constitution type as determined by pulse diagnosis. FD symptoms and quality of life were assessed using the Nepean Dyspepsia Index-Korean version (NDI-K) and Functional Dyspepsia-Quality of Life (FD-QoL) scores before and 2 and 4 weeks post-treatment. Adherence to treatment and adverse events were also evaluated.

**Results:**

All three patients showed significant improvements in NDI-K and FD-QoL scores after 4 weeks of treatment. No adverse events were observed during the treatment period. In particular, one patient experienced temporary symptom relapse due to poor dietary adherence, suggesting that the effectiveness of ECD may be associated with adherence to the prescribed diet.

**Conclusion:**

ECA combined with ECD led to significant symptom improvement and improved the quality of life in patients with chronic FD. These findings support the potential use of ECM-based interventions as effective and personalized treatment approaches for FD. More studies with larger sample sizes and standardized tools to assess diet adherence are required to validate these results and explore long-term outcomes.

## 1 Introduction

Functional dyspepsia (FD) is a common functional disorder with a prevalence of 10–20% worldwide. In South Korea, approximately 5% of the patients visiting primary care clinics are diagnosed with dyspepsia (1). Of those referred to tertiary care, 70–92% are diagnosed with FD, highlighting its significance in primary care (2). From 2019 to 2022, dyspepsia was ranked as the eighth most common diagnosis among outpatients at Korean medical institutions, being the only internal medicine-related condition among the top ten (3).

Functional dyspepsia is diagnosed according to the Rome IV criteria (4); however, despite the standardized diagnostic definitions, the assessment of the severity of the symptoms remains ambiguous. The lack of specific biomarkers for FD complicates its diagnosis, as it was based heavily on patient-reported symptoms. Moreover, symptoms overlap with those of other gastrointestinal disorders, fueling the ongoing debate regarding diagnosis of FD (5). While factors such as gastric motility disorders, vagal dysfunction, *Helicobacter pylori* infection, visceral hypersensitivity, and psychological components have been identified, the correlation between these pathophysiological findings and patient symptoms remains unclear, making treatment challenging. Moreover, the treatment of FD has largely remained empirical, with current drug therapies being limited and primarily symptom based (6). Therefore, there remains a significant unmet need to improve FD using current pharmacological therapies, as the side effects associated with drug treatments pose challenges for both clinicians and researchers.

Drugs, such as cisapride, have been associated with cardiac side effects, whereas metoclopramide has been associated with neurological side effects. Various pharmacological treatments exist, but network meta-analyses have reported that the efficacy of proton pump inhibitors (PPI) such as rabeprazole is modest, with symptom improvement in only 45–50% of patients (7, 8). Some studies have reported that mosapride shows no significant difference from placebo, while other studies have reported a placebo response rate of 30–40%, making it difficult to assess the actual efficacy of drug treatment. This variability in the effectiveness of pharmacological treatments for FD illustrates the challenges in treating the condition, as many patients experience only partial or short-term symptom relief, indicating the need for continuous management strategies. Recurrence rates are reported to be approximately 50% and those who have suffered from FD for more than 5 years are likely to have a worse prognosis (9). FD significantly affects quality of life and can cause psychiatric conditions such as depression. This also leads to a 40% increase in healthcare costs as patients require ongoing symptom management and additional diagnostic and therapeutic services (10).

Given these challenges, the demand for complementary and alternative medical approaches has increased, and acupuncture is receiving attention due to its clinical improvements in FD (11). It is known that FD is caused by mild inflammation of the stomach and duodenum, damage to the mucosa due to acid exposure, and dysfunction of the gut-brain axis, which disrupts the connectivity of the brain network and results in various patterns of symptoms. Acupuncture, a non-invasive method for neural stimulation, has been suggested to regulate gastrointestinal inflammation, inhibit acid secretion, and modulate the gut-brain axis through the microbiome (12). Many studies have investigated the efficacy of acupuncture in FD, with systematic reviews reporting that acupuncture achieves better symptom relief than conventional treatments, especially by modulating gut function through neural stimulation. These studies demonstrate the increasing use of acupuncture in clinical practice as an important complementary therapy for FD (13).

For centuries, both Western and Eastern medicine have recognized the importance of individual differences in medical approaches (14). The advent of precision and personalized medicine has further highlighted the significance of constitutional differences in managing chronic diseases (15). In East Asian traditional medicine, efforts to interpret individual differences have led to the development of various pattern identification systems (16). One such system, Eight Constitution Medicine (ECM), was first founded by Dr. Kuon Do-won in the 1960s. While sharing some philosophical roots with Traditional East Asian Medicine (TEAM), ECM independently emphasizes physiological organ dominance to define eight distinct constitutions. It integrates and newly reinterprets meridian theory, Epinger and Hess’s autonomic nervous system variability theory, and core concepts from traditional Korean Sasang medicine, including organ size disparity (臟腑大小) and functional organ dominance (臟腑機能偏性) (17).

Unlike general manual or electro-acupuncture treatments, Eight Constitution Acupuncture (ECA) follows constitution-specific formulas composed of fixed acupoint selections and sequences. These prescriptions are based on a unique meridian theory that distinguishes sending and receiving channels, and the procedure strictly applies a standardized shallow needling technique (1–3 mm at ≤45°) without needle retention (18). Eight constitution dietary therapy (ECD) prescribes different dietary approaches based on the patient’s constitution (17, 18). According to Choi (15), 47 ECM studies —ranging from randomized controlled trials to case reports—have been published, with most case reports demonstrating improvements in VAS scores or complete symptom resolution following ECA treatment (19). Moreover, recent systematic reviews on ECM provide evidence supporting ECM treatments (20, 21). Studies on ECM’s diagnostic methods indicate constitution-dependent gene expression phenotypes (22), and the standard pulse diagnosis method has also demonstrated validity (23). Furthermore, significant differences in the prevalence of metabolic syndrome have been reported among the eight constitutions (24). Although these findings offer supportive evidence for ECM-based treatments, the overall quality and numbers of the evidence remain limited. Notably, there have been no clinical studies on its application in improving FD. Although many studies have shown the efficacy of general manual acupuncture and herbal prescriptions for FD (25, 26), no research has been conducted on a standardized ECA for FD.

ECM has strengths in managing health through personalized treatment, emphasizing lifestyle and diet therapy. Therefore, this preliminary case series describes three patients with FD who experienced improvements in symptoms and quality of life scores after receiving ECA treatment. Due to the small sample size and absence of a control group, the generalizability of findings is limited. However, this study aims to generate hypotheses by presenting clinical outcomes and evaluating the potential of ECA and ECD for FD as a foundation for future controlled studies.

## 2 Methods

### 2.1 Participants and diagnosis

This study retrospectively selected three patients with long-standing FD who visited two primary Korean medicine clinics, A and B, between the second half of 2022 and the first half of 2023. This study aimed to analyze the effects of ECA and ECD in these patients. All patients were diagnosed with FD according to the Rome IV criteria, had received at least seven sessions of ECA, had no other underlying gastrointestinal diseases or history of medication use, and had not taken any dyspepsia-related drugs from other medical institutions in the past 3 months.

### 2.2 Ethical consideration

This retrospective case report includes the medical records of three patients diagnosed with FD. Written informed consent was obtained from the three patients and the study was approved by the Institutional Review Board (IRB) of Wonkwang University (approval number: WKIRB-202407-BM-036). Furthermore, this report adhered to the CARE guidelines for case reports (Supplementary Table 1).

### 2.3 Intervention

**Figure 1:**
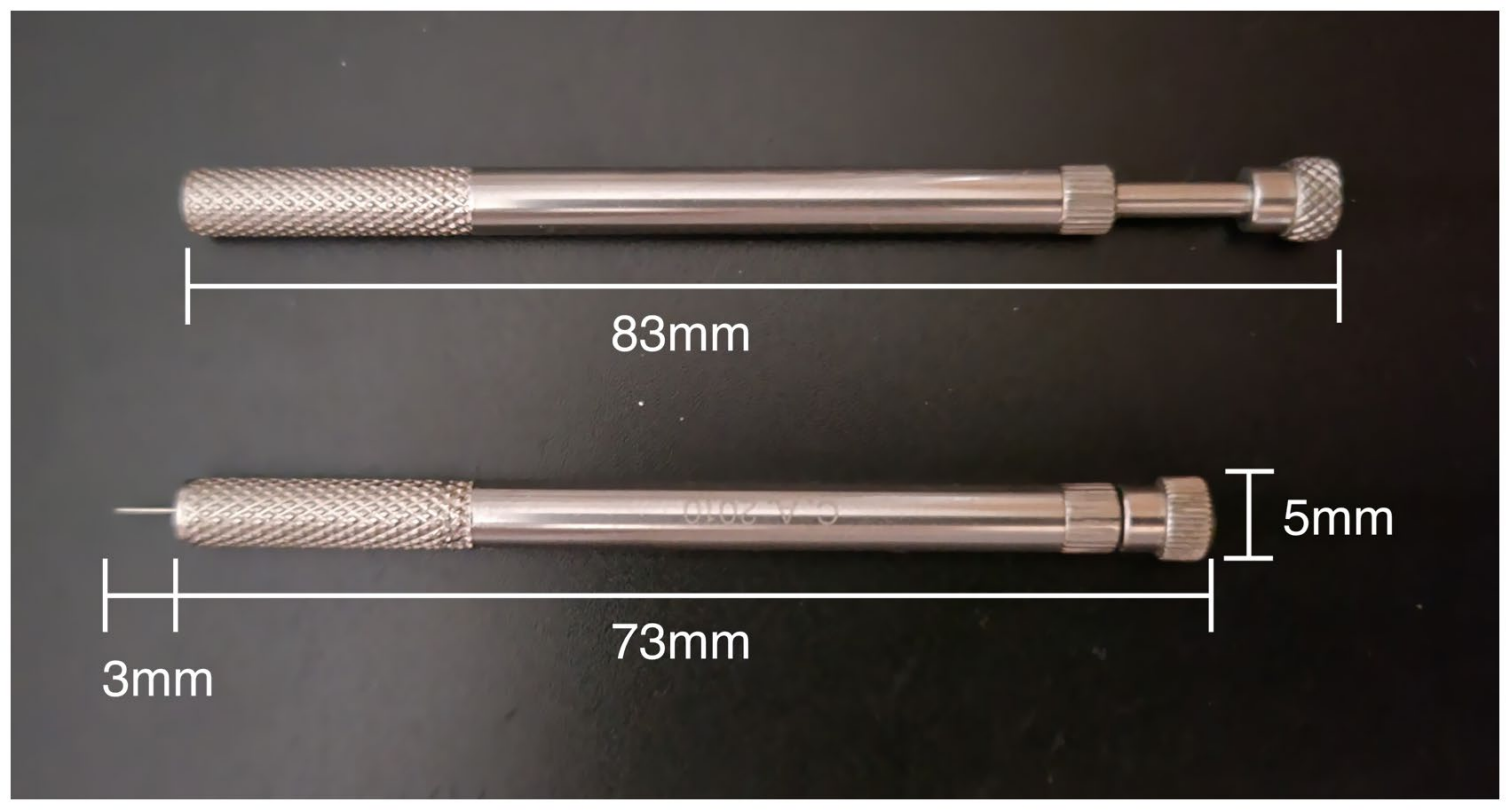
Standard eight constitution acupuncture guide tube.

ECA was administered by licensed Korean medicine doctors with more than 10 years of clinical experience in treating patients using ECM. Treatment involved the use of disposable stainless steel needles (Dongbang Medical, Korea) with a diameter of 0.25 mm and a length of 30 mm. The ECA method is a non-retention “fast-in-fast-out” method using a standard ECA needle guide tube (Sujin Medical, Korea) (Figure 1). A video of the standard ECA needle technique is provided in Supplementary File 1. Each patient’s constitutional type was determined using a standardized ECM pulse diagnostic technique. This method evaluates the depth, width, and strength of the radial artery pulse, allowing classification into one of the eight constitutional types (22, 23). In other words, treatment is not conducted using a single protocol, but rather tailored to each individual based on their constitutional type, with a distinct protocol applied accordingly. Detailed diagnostic criteria and treatment protocols for each constitution are presented in Supplementary File 2. After determining the patient’s condition and symptoms, ECA were administered based on standardized ECM treatment protocols and the treatment details were recorded in the patient’s medical chart. The selection of acupuncture points was consistent with ECM textbooks, specifically “biofunctional medicine” (27).

For the ECD, patients were educated about food avoidance and consumption according to their specific constitution (24). The dietary recommendations were provided in printed form, and either a specialized ECM practitioner or a trained research assistant explained the ECD guidelines to the patients.

### 2.4 Assessment

To evaluate changes in symptoms and quality of life before and after treatment, two scales were used: the Nepean Dyspepsia Index-Korean version (NDI-K) and the Functional Dyspepsia-Quality of Life (FD-QoL) questionnaire. The NDI-K is a dyspepsia-specific quality of life assessment tool that consists of symptom scoring and quality of life-related questions, with higher scores indicating a lower quality of life (28). The FD-QoL comprises 21 items that assess the quality of life in four categories: diet, daily activities, emotions, and social functioning. Each item is rated on a 5-point Likert scale (29). These assessments were administered through an online survey tool, with response links sent to patients’ smartphones. The results were evaluated by the same Korean medicine doctor who performed the ECM treatments. Scores were collected at the initial visit and at the 2- and 4-week follow-up visits. Adherence to ECD (eight constitution diet adherence, ECDA) was also assessed at the 2- and 4-week follow-up by asking patients to estimate their percentage of compliance, which was recorded in electronic medical records.

To assess subjective symptoms, we used electronic patient-reported outcomes (ePRO) as the primary outcome measure. ePRO is an accurate and systematic tool for evaluating subjective symptoms such as dyspepsia, helping minimize data loss in primary care settings through smartphone-based response collection. The use of ePRO is particularly recommended for treatments such as ECM, which emphasizes lifestyle and dietary management, and should be adopted more widely in clinical practice for a complete evaluation of symptoms. To evaluate the safety of ECA and ECD, adverse reactions during treatment were assessed through patient interviews at each visit and recorded on medical records.

### 2.5 Case presentation

#### 2.5.1 Case 1

A 52-year-old male patient presented epigastric tightness, abdominal bloating, involuntary bowel sounds, and diarrhea that occurred three times a week. He had visited the emergency department for gastric spasms in February 2023 and, after recognizing the severity of his symptoms, sought treatment at Clinic A. His previous esophagogastroduodenoscopy (EGD) results were normal and he had been diagnosed with irritable bowel syndrome, self-medicating with antidiarrheal agents, gastrointestinal motility regulators, and antacids as needed. At the time of his visit, he was taking omega-3, lutein, and multivitamin supplements. Upon diagnosis, the patient was classified as having Colonotonia (Metal Yin Constitution) according to the ECM pulse diagnosis, and ECA and ECD treatments were initiated.

At the start of treatment, he presented with diarrhea, upper abdominal bloating, and bothersome early satiation due to dyspepsia. His initial NDI-K and FD-QoL scores were 79 and 27, respectively. Over a period of approximately 10 months, the patient received 31 ECA sessions, initially two to three times a week, which was later reduced to once every 1–2 weeks as symptoms stabilized. His self-reported adherence to ECD was 90% for the first 2 weeks and maintained at 90% for the next 2 weeks.

**Figure 2:**
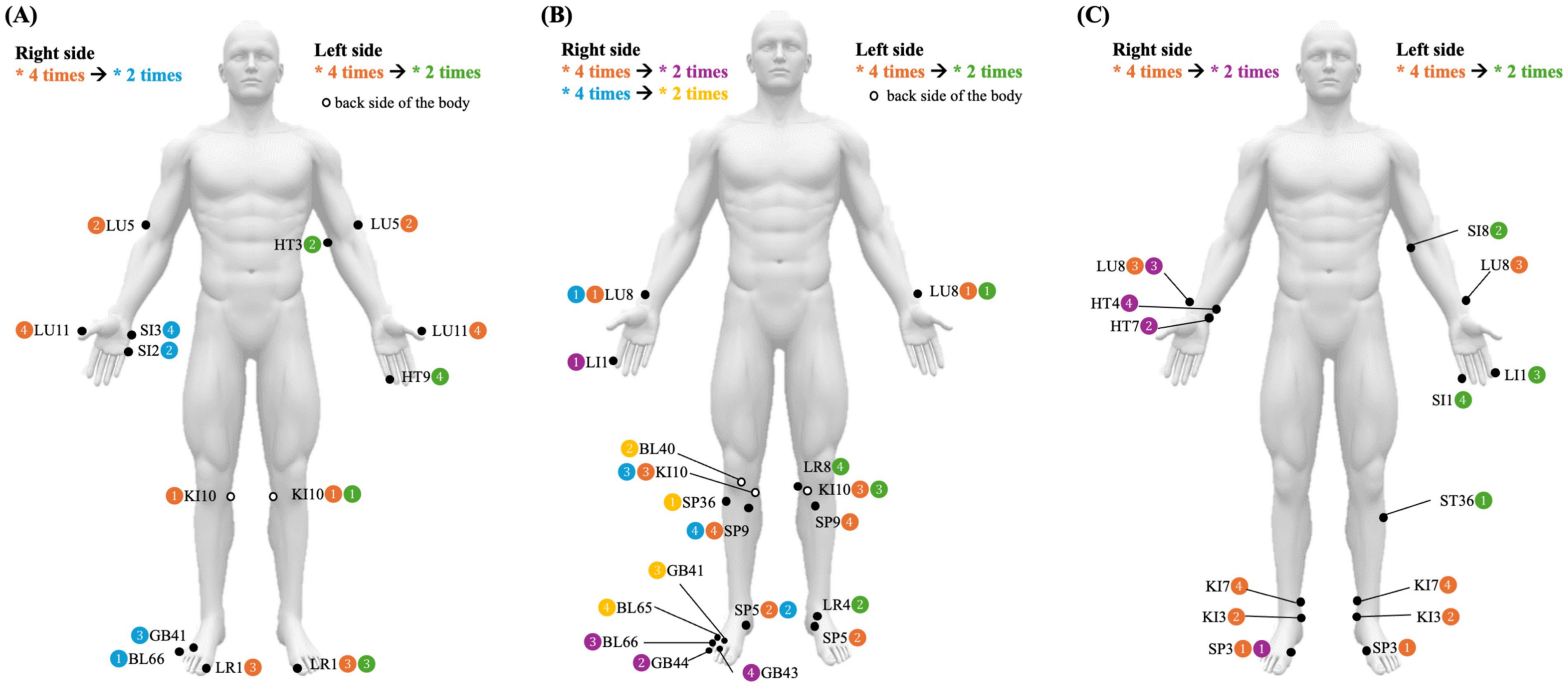
Schematic diagrams of the acupuncture points used for each patient. **(A)** Case 1. **(B)** Case 2. **(C)** Case 3.

According to his Colonotonia constitution and symptoms, the primary acupuncture prescription for each visit was ‘COL VIIq III,/VIIq IV,’ ‘COL VIIq III,’ was the main prescription targeting gastrointestinal inflammation, known as the “anti-inflammatory prescription.” Although ‘COL VLLq IV,’ was an auxiliary prescription to inhibit abnormal bacteria in inflammatory conditions, known as the “bactericidal prescription” (Table 1 and Figure 2). For ECD, the patient was advised to avoid meat and flour-based foods and was particularly restricted from eating ginger, garlic, apples, pears, melons, coffee, nuts, and mushrooms. Seafood, leafy green vegetables, strawberries, peaches, cherries, persimmons, grapes, pineapples, and cocoa are recommended.

**Table 1:** Eight constitution acupuncture formula of three cases.

Case no.	ECA formula	Therapeutic effect	ECA therapeutic process
1	COL VIIq III, (Left side)	Anti-inflammatory	Step 1 IX’9a(KI10) - > VII’9a(LU5) - > I’1c(LR1) - > VII’1c(LU11) – Repeat four times in sequence. Step 2 IX’9a(KI10) - > III’9a(HT3) - > I’1c(LR1) - > III’1c(HT9) – Repeat two times in sequence.
	COL VIIq IV, (Right side)	Bactericidal	Step 1 IX’9a(KI10) - > VII’9a(LU5) - > I’1c(LR1) - > VII’1c(LU11) – Repeat four times in sequence. Step 2 X’10a(BL66) - > IV’10a(SI2) - > II’2c(GB41) - > IV’2c(SI3) – Repeat two times in sequence.
2	VES Vq I, (Left side)	Anti-inflammatory	Step 1 VII’7c(LU8) - > V’7c(SP5) - > IX’9a(KI10) - > V’9a(SP9) – Repeat four times in sequence. Step 2 VII’7c(LU8) - > I’7c(LR4) - > IX’9a(KI10) - > I’9a(LR8) – Repeat two times in sequence.
	VES Vq II, (Right side)	Bactericidal	Step 1 VII’7c(LU8) - > V’7c(SP5) - > IX’9a(KI10) - > V’9a(SP9) – Repeat four times in sequence. Step 2 VIII’8c(LI1) - > II’8c(GB44) - > X’10a(BL66) - > II’10a(GB43) – Repeat two times in sequence.
	VES Vq X, (Right side)	Vitalizing	Step 1 VII’7c(LU8) - > V’7c(SP5) - > IX’9a(KI10) - > V’9a(SP9) – Repeat four times in sequence. Step 2 VI’6c(ST36) - > X’6c(BL40) - > II’2a(GB41) - > X’2a(BL65) – Repeat two times in sequence.
3	PAN IXq III, (Right side)	Anti-inflammatory	Step 1 V’5a(SP3) - > IX’5a(KI3) - > VII’7c(LU8) - > IX’7c(KI7) – Repeat four times in sequence. Step 2 V’5a(SP3) - > III’5a(HT7) - > VII’7c(LU8) - > III’7c(HT4) – Repeat two times in sequence.
	PAN IXq IV, (Left side)	Bactericidal	Step 1 V’5a(SP3) - > IX’5a(KI3) - > VII’7c(LU8) - > IX’7c(KI7) – Repeat four times in sequence. Step 2 VI’6a(ST36) - > IV’6a(SI8) - > VIII’8c(LI1) - > IV’8c(SI1) – Repeat two times in sequence.

The selection of the acupuncture point and the treatment sequence for each case were documented. Once the ECA prescription formula is determined, the treatment efficacy is individualized, and the acupuncture point selection and treatment sequence are established accordingly. COL, Colonotonia; VES, Vesicotonia; PAN, Pancreotonia; q, symbol of 4-time needling; symbol of 2-time needling; VII, III, IV, V, I, II, X, IX, number of organs; V’, VII’, IX’, I′, VIII’, II’, X’, number of the meridians; 1, 2, 5, 6, 7, 8, 9, 10, number of acupoints; a, anapuncture (con-puncture); c, cata-puncture (pro-puncture).

**Figure 3:**
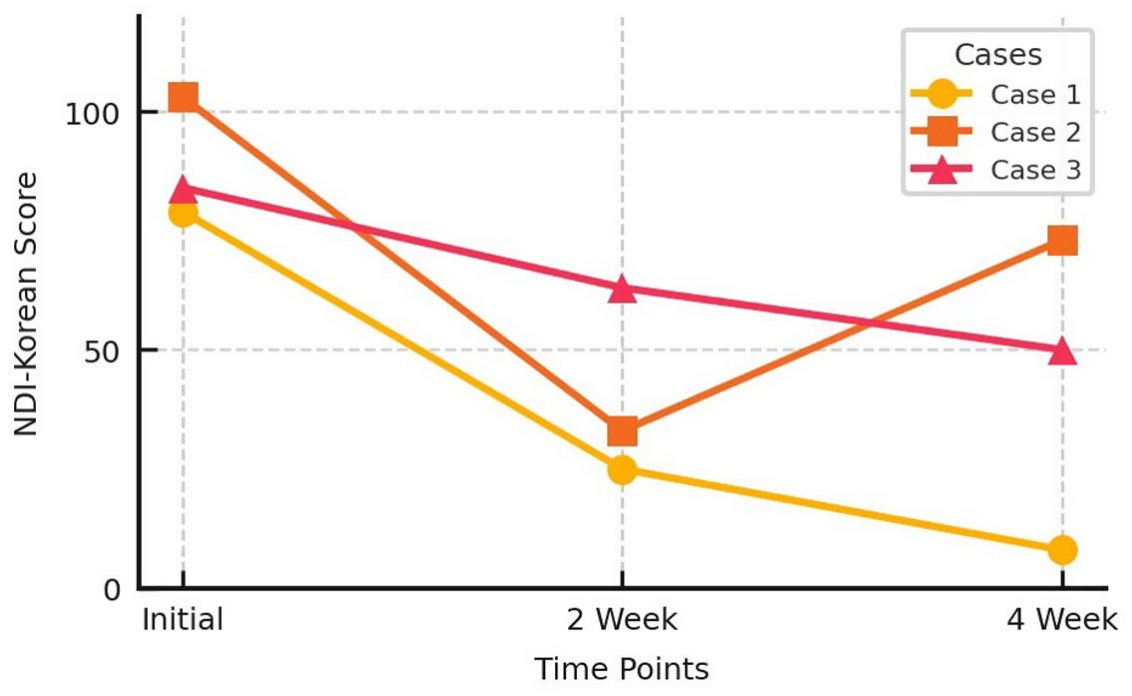
Nepean Dyspepsia Index-Korean version score change of three cases. NDI, Nepean Dyspepsia Index.

**Figure 4:**
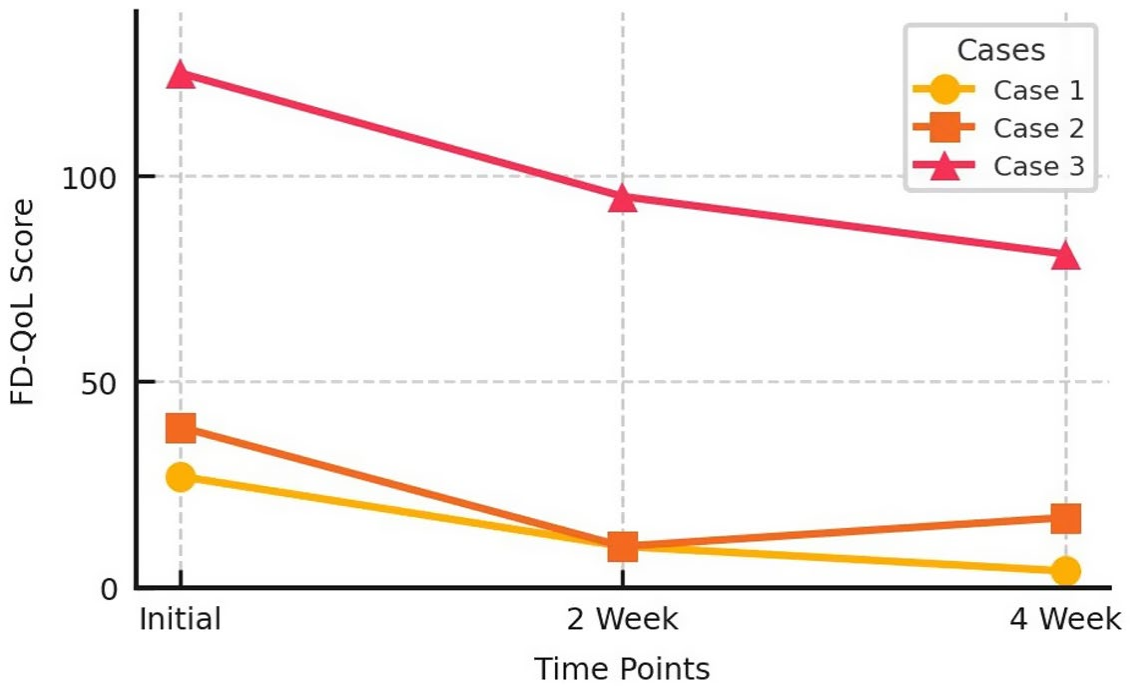
Functional Dyspepsia-Quality of Life score change of three cases.

Two weeks after treatment, the patient’s NDI-K score improved to 25, and his FD-QoL score improved to 10. After 4 weeks, his NDI-K and FD-QoL scores improved further to 8 and 5, respectively. No adverse effects were observed during treatment (Figures 3, 4).

#### 2.5.2 Case 2

A 34-year-old female patient had experienced persistent fullness below the sternum, frequent belching, and lower abdominal gas for the past 5–6 years, requiring her to take digestive medication one to two times a week. She commuted to work by bus for 1.5 h daily for the last 9 years, suffering from severe motion sickness and constantly cold hands and feet. Despite repeated visits to various internal medicine and Korean medicine clinics, her symptoms persisted, and her gastroscopy results were consistently normal. She visited Clinic B with these symptoms, where she was diagnosed with Vesicotonia (Water Yin Constitution) and began treatment with ECA and ECD.

At the beginning of treatment, her NDI-K and FD-QoL scores were 103 and 39, respectively. Over a 4-week treatment period, she received seven ECA sessions, with treatments targeting gastritis for the first two sessions and then focused on gastrointestinal activation for the next five sessions. Adherence to ECD was self-reported to be 60% during the first 2 weeks and 70% during the next 2 weeks.

According to Vesicotonia constitution and symptoms, ECA administered at each visit first aimed to treat gastrointestinal inflammation, followed by activation of the gastrointestinal muscles. The standard ECA prescriptions used were ‘VES Vq I, / VIIq II,’ and ‘VES Vq I, / Vq X,’ ‘Vq I,’ is the main prescription in Vesicotonia for treating gastrointestinal inflammation, known as the “anti-inflammatory prescription,” while ‘Vq II,’ is an auxiliary prescription used to suppress abnormal bacteria in inflammatory conditions, referred to as the “bactericidal prescription.” ‘Vq X,’ is another auxiliary prescription aimed at activating weakened gastrointestinal muscles, called the “vitalizing prescription” (Table 1 and Figure 2). In ECD education for patients with Vesicotonia, cold foods were particularly restricted and the patient was advised to avoid coffee, seafood, persimmons, melons, bananas, strawberries, and green grapes. Recommended foods include chicken, beef, chili peppers, ginger, scallions, apples, tangerines, oranges, mangoes, and tomatoes.

Two weeks after starting treatment, the patient reported complete disappearance of symptoms, with reduced motion sickness and warmer hands and feet. Her NDI-K score improved to 33 and her FD-QoL score was 10. However, after experiencing significant improvement, she reverted to her old habits of drinking ice coffee and overeating, causing a slight recurrence of symptoms. By the fifth week, her NDI-K score had worsened to 74 and her FD-QoL score to 17, although both scores much better than her score on initial visit. No adverse effects were observed during treatment (Figures 3, 4).

#### 2.5.3 Case 3

A 52-year-old female patient had been experiencing discomfort below the sternum, constant bloating, abdominal gas, and nausea for 9 months, with symptoms worsening in the morning. Her symptoms fluctuated in severity but worsened 1 to 2 weeks before her visit. At the time of symptom onset, she was taking various supplements, including selenium, calcium, omega-3, eye supplements, vitamin B, and Chimhyanghwan (a Korean herbal supplement). However, her symptoms worsened after taking digestive medications to relieve them. No abnormalities were observed despite testing and treatment in a local internal medicine clinic. She was diagnosed with Pancreotonia (Earth Yang Constitution) in Clinic B and underwent ECA and ECD treatments.

At the start of treatment, her NDI-K score was 84 and her FD-QoL score was 125. Over a 3-month period, the patient underwent 29 ECA sessions, initially two to three times a week. Adherence to ECD was self-reported to be 80% for the first 2 weeks and 90% for the next 2 weeks.

For Pancreotonia, the primary ECA prescription was “PAN IXq III, / IXq IV,” “IXq III,” is the main auxiliary prescription targeting gastrointestinal inflammation, known as the “anti-inflammatory prescription,” while “IXq IV,” is an auxiliary prescription aimed at suppressing abnormal bacteria activated in inflammatory conditions, known as the “bactericidal prescription” (Table 1, Figure 2). For ECD, the patient was advised to avoid spicy seasonings, such as chili, ginger, and scallions, as well as chicken, lamb, brown rice, apples, oranges, mangoes, and tomatoes. Recommended foods include seafood, pufferfish, pork, beef, vegetables, persimmons, pears, melons, strawberries, and bananas.

After 2 weeks of treatment, the patient’s NDI-K score improved to 63 and her FD-QoL score improved to 95. After 4 weeks, her NDI-K score improved further to 50, and her FD-QoL score was 81 (Figures 3, 4). No adverse effects were reported during the treatment period.

## 3 Discussion

This study reports the effects of ECA and ECD sessions in three patients with FD. These three patients had long-standing dyspepsia for which they had undergone repeated treatments, including acupuncture, herbal medicine, and pharmacological therapies, without significant improvement. Furthermore, poor diet management leads to repeated cycles of improvement and relapse. Three patients with different constitutions received tailored ECA prescriptions and ECD guidelines. The cases were selected without prior knowledge of the treatment outcomes to prevent selection bias. After 4 weeks, the three patients showed significant subjective improvements in symptoms and quality of life. No adverse events were observed during the treatment period. Improvement in these symptoms is difficult to objectively verify and can only be measured based on subjective symptoms. Therefore, it was evaluated using tools, such as the NDI-K and FD-QoL, which are part of the ePRO system that has been increasingly used in recent research and patient-centered clinical practice (30).

In Case 2, the patient maintained a high level of adherence to ECD for 2 weeks after receiving ECA treatment, resulting in rapid improvement. However, after 2 weeks, the patient became complacent and reverted to her old habits, such as consuming iced coffee, leading to worsening of symptoms and quality of life scores at week 4. In contrast, the patients in Cases 1 and 3 maintained a high level of dietary adherence (self-reported) throughout the 4 weeks, resulting in a continued improvement in their symptoms, as confirmed by the PROs at weeks 2 and 4. Although dietary interventions are generally known to alleviate gastrointestinal symptoms, the constitution-specific nature of ECD may offer a more individualized approach. In this study, each patient received a different dietary protocol tailored to their constitution, however, all three showed clinical improvement. Notably, Cases 2 and 3 followed entirely opposite dietary recommendations, but both experienced symptom relief, suggesting that the therapeutic effects may not simply be attributed to generic diet modification. Patients with higher adherence to ECD appeared to experience more substantial improvements, indicating a potential link between compliance and treatment efficacy. However, since ECA and ECD were administered concurrently, it is difficult to differentiate the independent effects of each modality. Further controlled studies are needed to evaluate whether different constitutional types respond differently to their respective ECD protocols and to clarify the distinct contributions of ECA and ECD in treating FD.

Although each patient’s constitution and dietary recommendations were different, as were their ECA prescriptions, all patients achieved the common goal of improving their NDI-K and FD-QoL scores after 4 weeks of treatment. Furthermore, the NDI-K’s Minimal Clinically Important Difference (MCID) was 10 points (31), and the NDI-K scores in these cases decreased by 71, 30, and 34 points, respectively, all of which exceeded the MCID threshold. These findings are consistent with the goals of personalized and precision medicine, as the same disease (FD) was treated with constitution-specific dietary and acupuncture treatments, and similar improvements were observed. Furthermore, these cases highlight the importance of lifestyle management to maintain symptom improvement, showing that Korean medicine doctors should not only focus on treatment, but also actively engage in post-treatment lifestyle management.

ECM-based ECA involves the selection of different acupuncture points for each constitution, even when treating the same disease. When diagnosed with the same constitution, the selection of acupuncture points is predetermined, allowing for a more standardized and consistent application compared with conventional acupuncture treatments. As practitioners become more experienced, this treatment protocol can be standardized to improve its reproducibility and reliability (32). ECM uses a standardized pulse diagnostic method to categorize patients into eight constitutions (22, 23). ECA is a minimally invasive “fast-in fast-out” acupuncture technique, where needles are inserted briefly and then removed. Conventional acupuncture has been shown to alleviate FD symptoms through mechanisms such as enhanced gastric motility, accelerated gastric emptying, autonomic nervous system regulation, gut-brain axis modulation, and reduced visceral hypersensitivity (12, 13). In addition to these mechanisms, ECA minimizes stimulation, enabling more targeted and efficient treatment. The precision of this method allows for targeted stimulation at specific depths and locations, thereby enhancing its effectiveness. The short insertion time and quick removal of the needle also reduce the risk of infection, making overall treatment safer. Furthermore, the simplicity and short duration of the procedure lead to fewer mild adverse reactions while providing superior results compared to conventional acupuncture, making it more acceptable to patients and easier to apply in clinical settings (33). In this case series, the mean differences of NDI-K and FD-QoL scores after 4 weeks were 45 and 29.67, respectively. A previous meta-analysis of randomized controlled trials on conventional acupuncture for FD reported a mean reduction in NDI scores of 20.91 points (95% CI: 6.55–35.26, *p* = 0.004), and an improvement in the Nepean Dyspepsia Life Quality Index of 10.49 points (95% CI: 0.24–20.74, *p* = 0.04) (34). These findings show consistency with previous research on conventional acupuncture for FD. Compared to general acupuncture or broad dietary recommendations, ECM-based approaches offer constitution-specific protocols that may enhance the degree of personalization. However, the current findings should be interpreted with caution due to the small sample size and absence of a control group.

In ECM, lifestyle management is emphasized with constitution-specific diet, bathing, and exercise methods. Each constitution has its own set of beneficial and harmful foods, and bathing and exercise methods differ according to constitution (27). Among these, ECD plays a central role in both prevention and treatment and is implemented in conjunction with ECA. Studies have shown that ECD can lead to positive changes in blood test results (35). Although there have been various studies on ECM, both domestically and internationally (21, 22), well-designed scientific research on its clinical efficacy remains insufficient (36). Findings from this case series indicate a possible association between adherence to the ECD approach and improvement in FD symptoms, providing a hypothesis that may inform the design of future clinical research.

This study was conducted in primary care settings to demonstrate how ECA and ECD can be applied in real-world clinical settings. Notably, it reports on three patients who shared the same diagnosis of FD but differed in their constitutional types, each receiving tailored treatments accordingly. All three patients showed clinical improvement, demonstrating the feasibility of personalized interventions based on ECM. Unlike a single-case report, this case series highlights the potential of constitution-based approaches across diverse patient profiles. In addition to symptom improvement, the study also assessed dyspepsia-related quality of life, offering a more comprehensive understanding of treatment impact. By applying constitution-specific interventions based on ECM, our study provides preliminary evidence that personalized approaches may address functional imbalances involved in the pathophysiology of FD. These findings support the potential of ECM as a framework for developing integrative treatment models that combine traditional and modern medical paradigms. Furthermore, the personalized nature of ECM interventions aligns with current trends in precision medicine, highlighting the need for further research integrating constitutional diagnostics with biomedical tools to optimize individualized treatment strategies for FD. By emphasizing patient-centered outcomes, this study provides a better understanding of the holistic effects of treatment. As these cases reflect patients from a Korean population, future studies should examine whether ECM classifications and responses differ across regions and ethnicities to improve generalizability.

In this study, adherence was evaluated using a self-reported percentage of compliance rather than objective methods such as diet records or biomarker analysis. To improve the accuracy of adherence assessment in future research, standardized tools like the ECDA questionnaire, as recommended by prior studies, should be employed (15, 37). The treatment effect was evaluated only using NDI-K and FD-QoL scores. Therefore, future studies are needed to include other objective indicators, such as gastroscopy or gastrointestinal motility tests. Moreover, this study only evaluated the effects over 4 weeks. To evaluate the long-term effects and sustainability of ECA and ECD, follow-up studies with long-term assessments are necessary. Although no adverse events were reported in this study, large-scale studies are required to comprehensively assess the safety of ECA and ECD. Given the diverse underlying causes of FD, further research is required to determine whether ECA and ECD are particularly effective for specific subtypes of FD or broadly applicable across etiologies. In the future, randomized controlled trials (RCTs) including sham acupuncture or waitlist control groups should be conducted to better evaluate the efficacy of ECA and ECD. Stratification by constitution type prior to randomization would help address individual variability. Standardized ECM-based treatment protocols and validated adherence measures, such as the ECDA questionnaire, should be utilized. In addition, incorporating objective outcomes, such as gastrointestinal motility tests and biomarker analysis, would enhance the scientific rigor of future research.

Furthermore, research on the physiological mechanisms underlying the differences between ECA and conventional acupuncture in the human body is lacking. While preliminary observations in this case series suggest symptom improvement, the exact mechanisms of ECA and ECD remain unclear. Prior research suggests that acupuncture may regulate gut-brain axis signaling, modulate gastrointestinal motility, and reduce inflammation, while dietary modifications can affect microbiota and immune function (12). However, how these mechanisms operate within ECM remains to be explored. This highlights the need for translational research integrating ECM with modern biomedical tools such as microbiome profiling, metabolomics, or neuroimaging, which could help validate the physiological mechanisms of ECM and enhance its scientific and clinical innovation.

## 4 Conclusion

This case report shows that combining ECA and ECD based on ECM may be associated with improvements in symptoms and quality of life in patients with FD. After 4 weeks of treatment, there was an improvement in the NDI-K and FD-QoL scores. No adverse events observed during the treatment period, suggesting a favorable safety profile. This study was designed as early-stage exploratory research aimed at reporting clinical cases and proposing hypotheses for future studies. Although it has limitations, including a small sample size, absence of a control group, and reliance on subjective outcomes, the consistent improvements observed across all cases suggest potential clinical benefits. Further well-designed clinical trials with larger populations and objective outcome measures are warranted to confirm these findings and evaluate long-term effectiveness.

## Data Availability

The raw data supporting the conclusions of this article will be made available by the authors, without undue reservation.
