# Supplementary material for: Case Report: Improvement of functional dyspepsia using eight constitution acupuncture and eight constitution diet – A report of three cases
Source: Front Med (Lausanne). 2025 Jul 23;12:1545687. doi: 10.3389/fmed.2025.1545687 (PMC12326475; doi:10.3389/fmed.2025.1545687)
Supplement: Supplementary file 1 [file Data_Sheet_1.docx]

**Supplementary Table 1.** CARE checklist.

**Title 1** The words “case report” should be in the title along with the area of focus title page

**Key Words 2** 2 to 5 key words that identify areas covered in this case report p.2

**Abstract 3a** Introduction—What is unique about this case? What does it add to the medical literature? p.1

**3b** The main symptoms of the patient and the important clinical findings p.1

**3c** The main diagnoses, therapeutics interventions, and outcomes p.1

**3d** Conclusion—What are the main “take-away” lessons from this case? p.2

**Introduction 4** One or two paragraphs summarizing why this case is unique with references p.4-5

**Patient Information 5a** Demographic information and other patient specific information p.8-11

**5b** Main concerns and symptoms of the patient p.8-11

**5c** Medical, family, and psychosocial history including relevant genetic information (also see timeline). p.8-11

**5d** Relevant past interventions and their outcomes p.8,10,11

**Clinical Findings 6** Describe the relevant physical examination (PE) and other significant clinical findings p.8-11

**Timeline 7** Important information from the patient’s history organized as a timeline N/A

**Diagnostic Assessment**

**Therapeutic Intervention**

**Follow-up and Outcomes**

**8a** Diagnostic methods (such as PE, laboratory testing, imaging, surveys) p.7

**8b** Diagnostic challenges (such as access, financial, or cultural) p.3

**8c** Diagnostic reasoning including other diagnoses considered p.6

**8d** Prognostic characteristics (such as staging in oncology) where applicable p.7

**9a** Types of intervention (such as pharmacologic, surgical, preventive, self-care) p.9-12, table 1, fig 1

**9b** Administration of intervention (such as dosage, strength, duration) p.9-12, table 1

**9c** Changes in intervention (with rationale) N/A

**10a** Clinician and patient-assessed outcomes (when appropriate) p.7

**10b** Important follow-up diagnostic and other test results p.9-12, fig 2,3

**10c** Intervention adherence and tolerability (How was this assessed?) p.9-11

**10d** Adverse and unanticipated events p.9,11,12

**Discussion 11a** Discussion of the strengths and limitations in your approach to this case p.16-17

**11b** Discussion of the relevant medical literature p.14-16

**11c** The rationale for conclusions (including assessment of possible causes) p.17

**11d** The primary “take-away” lessons of this case report p.17

**Patient Perspective 12** When appropriate the patient should share their perspective on the treatments they received N/A

**Informed Consent 13** Did the patient give informed consent? Please provide if requested . . . . . . . . . . . . . . . . . . . . . . . . . . . . . . . . . . . . . . p.6

Supplementary **File 1.** Instructions and demonstration video for using the standard eight constitution acupuncture guide tube.

ECM Study Kor [Internet]. ECA method: 2024 Available from: <https://youtu.be/kgJ4tFryM0s>

**
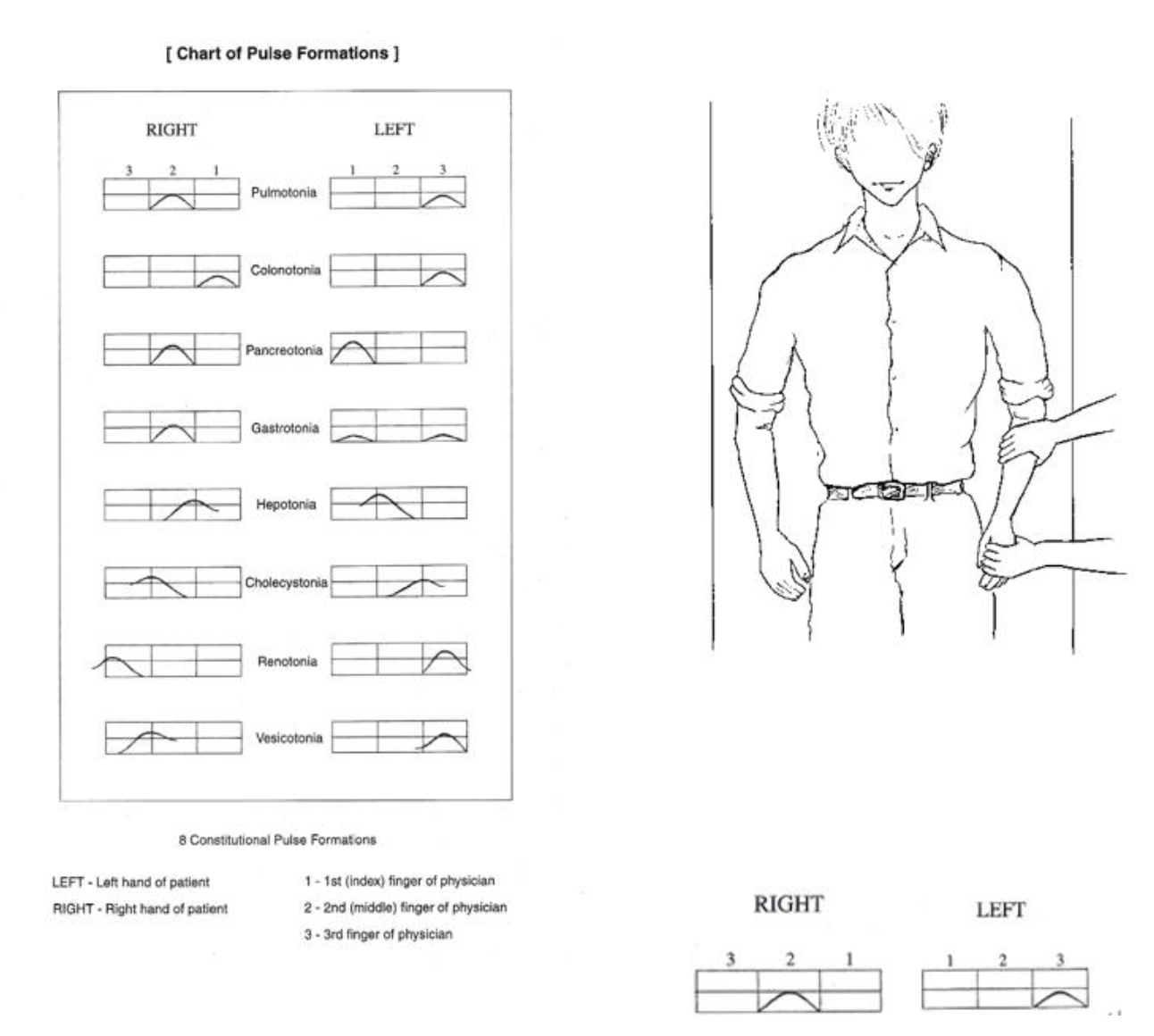
Supplementary File 2. Eight constitution medicine protocol**

**A. Eight pulse patterns of the radial artery in eight constitution medicine**: ECM pulse diagnosis is different from the traditional Korean pulse diagnosis, in terms of the location at which it is performed and the method that is used. The diagnostic procedure is as follows: First, the physician asks the patient to lie down and grips the patient's LEFT wrist (LEFT) with the doctor's right three fingers contacting the patient's radial artery. The position of the physician's index finger (1), middle finger (2) and ring finger (3) is placed on the radial artery line that is below the patient’ s radial styloid process. Next, the physician presses the patient's radial artery until the pulse can no longer be felt. Then, the physician releases the pressure of three fingers applied to the artery and catches where the first strongest pulse bounces up. The same process is also performed on the patient's right wrist with the physician's left hand (RIGHT). The physician should take into consideration the inclination of the patient's radius and give the three fingers the same strength to press the patient's wrist evenly. The diagnosis of eight constitutions were made using unique pulse patterns composed of the combination of pulse formations on both the left and right radial arteries. The curved line in the present figure indicates the first pulse wave increase; the box indicates the pulse strength. (1,2)

**B. Principles and Physiological Basis of Pulse Diagnosis in Eight Constitution Medicine (ECM)**

Pulse diagnosis in Eight Constitution Medicine (ECM) is a critical method used to determine a patient's constitutional type. This technique involves assessing the depth, width, and strength of the radial pulse at both wrists. Diagnosis is based on the asymmetric distribution of strength between paired organ systems (e.g., lungs vs. liver, kidneys vs. heart), reflecting the patient's innate functional hierarchy of organs, which is a core principle of ECM.(3,4)

The pulse signals evaluated in ECM are believed to reflect autonomic nervous system (ANS)-mediated activity and regulatory dynamics of internal organs, which are also associated with immune balance and inflammatory responses. Beyond traditional pulse assessment, ECM integrates the concept of ANS regulation, particularly the balance between sympathetic and parasympathetic dominance.(1) Dr. Dowon Kuon, the founder of ECM theory, classified eight distinct pulse patterns based on clinical experience, considering the pulse to represent the flow of all internal organ functions. The theoretical foundation of ECM pulse diagnosis posits that pulse characteristics mirror ANS-mediated organ functions, offering insights into constitutional imbalances that predispose individuals to specific pathological conditions.

For example, in the Hepatonia (Wood Yang Constitution), the organs are functionally dominant in the order of liver, kidney, heart, spleen, and lung. According to ECM theory, such organ asymmetry does not necessarily indicate disease but rather reflects constitutional predispositions that influence disease susceptibility and treatment responses. It is proposed that inappropriate lifestyle habits or dietary patterns, misaligned with one's constitution, can disrupt this balance, potentially leading to organ dysfunction and the manifestation of disease symptoms.

Recent studies have supported the physiological validity of this diagnostic system. For instance, Kim et al.(2), reported that individuals classified into different ECM constitutions exhibited distinguishable trends in gene expression profiles and organ-specific metabolic patterns, such as relative dominance in lung or liver phenotypes. These findings tentatively suggest that constitutional types determined through pulse assessment could be associated with underlying biological and physiological characteristics, although further validation is needed.

In terms of reproducibility, Shin et al.(5) observed a inter-rater reliability among practitioners employing ECM pulse diagnosis. The application of standardized finger positioning, controlled pressure, and systematic mapping of pulse areas may contribute to improving diagnostic consistency and clinical applicability.

Taken together, while preliminary findings provide some support for the physiological relevance and reproducibility of ECM pulse diagnosis, further rigorous research is required to substantiate its reliability and clinical significance.

| Constitution Type | Dominant Organs | Autonomic Nervous System Tendency |
| --- | --- | --- |
| Hepatonia | Liver > Kidney > Heart > Small Intestine > Lung | Parasympathetic > Sympathetic |
| Cholecystonia | Gallbladder > Small Intestine > Stomach > Bladder > Large Intestine | Parasympathetic > Sympathetic |
| Pancreotonia | Pancreas > Heart > Liver > Lung > Kidney | Parasympathetic > Sympathetic |
| Gastrotonia | Stomach > Large Intestine > Small Intestine > Gallbladder > Bladder | Parasympathetic > Sympathetic |
| Pulmotonia | Lung > Pancreas > Heart > Kidney > Liver | Sympathetic > Parasympathetic |
| Colonotonia | Large Intestine > Bladder > Stomach > Small Intestine > Gallbladder | Sympathetic > Parasympathetic |
| Renotonia | Kidney > Lung > Liver > Heart > Pancrea | Sympathetic > Parasympathetic |
| Vesicotonia | Bladder > Gallbladder > Small Intestine > Large Intestine > Stomach | Sympathetic > Parasympathetic |

C**. ECA prescription for each constitution (1)**

| 74* | ECA formula | ECA therapeutic process (According to Needle Sequence) | | | ECA formula | ECA therapeutic process (According to Needle Sequence) | ECA formula | ECA therapeutic process (According to Needle Sequence) | | ECA formula | ECA therapeutic process (According to Needle Sequence) | |
| --- | --- | --- | --- | --- | --- | --- | --- | --- | --- | --- | --- | --- |
| EC |  | **Pulmotonia VII(VIII) > V(VI) >III(IV) > IX(X) > I(II)** | | |  | **Hepatonia I(II) > IX(X) > III(IV)**  **> V(VI) > VII(VIII)** | **EC** | **Colonotonia**  **Ⅶ(Ⅷ)>Ⅸ(Ⅹ)>Ⅴ(Ⅵ)**  **>Ⅲ ( Ⅳ ) >Ⅰ ( Ⅱ )** | |  | **Cholecystonia**  **Ⅰ ( Ⅱ ) >Ⅲ ( Ⅳ ) > Ⅴ(Ⅵ)**  **> Ⅸ(Ⅹ)> Ⅶ(Ⅷ)** | |
| F* | I'p** (Promote) | VII'7a** I'7a IX'9c** I'9c LU8 LR4 KI10 LR8 | | | I's** (Suppress) | VII'7c I'7c IX'9a I'9a LU8 LR4 KI10 LR8 | Ⅶs | Ⅸ'9a Ⅶ'9a Ⅰ'1c Ⅶ'1c  KI10 LU5 LR1 LU11 | | Ⅶp | Ⅸ'9c Ⅶ'9c Ⅰ'1a Ⅶ'1a  KI10 LU5 LR1 LU11 | |
|  | II'p | VIII'8a II'8a X'10c II'10c LI1 GB44 BL66 GB43 | | | II's | VIII'8c II'8c X'10a II'10a LI1 GB44 BL66 GB43 | Ⅷs | Ⅹ'10a Ⅷ'10a Ⅱ'2c Ⅷ'2c  BL66 LI2 GB41 LI3 | | Ⅷp | Ⅹ'10c Ⅷ'10c Ⅱ'2a Ⅷ'2a  BL66 LI2 GB41 LI3 | |
|  | IX'p | V'5a IX'5a I'1c IX'1c SP3 KI3 LR1 KI1 | | | IX's | V'5c IX'5c I'1a IX'1a SP3 KI3 LR1 KI1 | Ⅸs | Ⅶ'7a Ⅸ'7a Ⅲ'3c Ⅸ'3c  LU8 KI7 HT8 KI2 | | Ⅸp | Ⅶ'7c Ⅸ'7c Ⅲ'3a Ⅸ'3a  LU8 KI7 HT8 KI2 | |
|  | X'p | VI'6a X'6a II'2c X'2c ST36 BL40 GB41 BL65 | | | X's | VI'6c X'6c II'2a X'2a ST36 BL40 GB41 BL65 | Ⅹs | Ⅷ'8a Ⅹ'8a Ⅳ'4 c Ⅹ'4c  LI1 BL67 SI5 BL60 | | Ⅹp | Ⅷ'8c Ⅹ'8c Ⅳ'4 a Ⅹ'4a  LI1 BL67 SI5 BL60 | |
| IF* | V's** | VII'7a V'7a IX'9c V'9c LU8 SP5 KI10 SP9 | | | V'p | VII'7c V'7c IX'9a V'9a LU8 SP5 KI10 SP9 | Ⅲp | Ⅸ'9a Ⅲ'9a Ⅰ'1c Ⅲ'1c  KI10 HT3 LR1 HT9 | | Ⅲs | Ⅸ'9c Ⅲ'9c Ⅰ'1a Ⅲ'1a  KI10 HT3 LR1 HT9 | |
| B* | VI's | VIII'8a VI'8a X'10c VI'10c LI1 ST45 BL66 ST44 | | | VI'p | VIII'8c VI'8c X'10a VI'10a LI1 ST45 BL66 ST44 | Ⅳp | Ⅹ'10a Ⅳ'10a Ⅱ'2c Ⅳ'2c  BL66 SI2 GB41 SI3 | | Ⅳs | Ⅹ'10c Ⅳ'10c Ⅱ'2a Ⅳ'2a  BL66 SI2 GB41 SI3 | |
| IZ* | VII's | V'5a VII'5a I'1c VII'1c SP3 LU9 LR1 LU11 | | | VII'p | V'5c VII'5c I'1a VII'1a SP3 LU9 LR1 LU11 | Ⅰp | Ⅶ'7a Ⅰ'7a Ⅲ'3c Ⅰ'3c  LU8 LR4 HT8 LR2 | | Ⅰs | Ⅶ'7c Ⅰ'7c Ⅲ'3a Ⅰ'3a  LU8 LR4 HT8 LR2 | |
| V* | VIII's | VI'6a VIII'6a II'2c VIII'2c ST36 LI11 GB41 LI3 | | | VIII'p | VI'6c VIII'6c II'2a VIII'2a ST36 LI11 GB41 LI3 | Ⅱp | ⅦI'8a Ⅱ'8a Ⅳ'4 c Ⅱ'4c  LI1 GB44 SI5 GB38 | | Ⅱs | ⅦI'8c Ⅱ'8c Ⅳ'4 a Ⅱ'4a  LI1 GB44 SI5 GB38 | |
| P* | III's | $\bar{\mathrm{III}}$'5a $\bar{\mathrm{III}}$'9c PC7 PC3 | | | $\bar{\mathrm{III}}$'p | $\bar{\mathrm{III}}$'5c $\bar{\mathrm{III}}$'9a PC7 PC3 | Ⅲ’p | Ⅲ'7a Ⅲ'1c  HT4 HT9 | | Ⅲ’s | Ⅲ'7c Ⅲ'1a  HT4 HT9 | |
|  | IV's | $\bar{\mathrm{IV}}$'6a $\bar{\mathrm{IV}}$'0c TE10 TE2 | | | $\bar{\mathrm{IV}}$'p | $\bar{\mathrm{IV}}$'6c $\bar{\mathrm{IV}}$'0a TE10 TE2 | Ⅳ’p | Ⅳ'8a Ⅳ'2c  SI1 SI3 | | Ⅳ’s | Ⅳ'8c Ⅳ'2a  SI1 SI3 | |
| EC |  | | **Pancreotonia**  **Ⅴ(Ⅵ)>Ⅲ ( Ⅳ ) >Ⅰ ( Ⅱ ) > Ⅶ(Ⅷ)>Ⅸ(Ⅹ)** |  | | **Renotonia**  **Ⅸ(Ⅹ)> Ⅶ(Ⅷ)>Ⅰ ( Ⅱ )**  **>Ⅲ ( Ⅳ ) >Ⅴ(Ⅵ)** |  | **Gastrotonia**  **Ⅴ(Ⅵ)>Ⅶ(Ⅷ)>Ⅲ ( Ⅳ ) >Ⅰ ( Ⅱ ) >Ⅸ(Ⅹ)** |  | | **Vesicotonia**  **Ⅸ(Ⅹ)>Ⅰ ( Ⅱ ) >Ⅲ ( Ⅳ ) >Ⅶ(Ⅷ)>Ⅴ(Ⅵ)** |  |
| F* | IXp | | V'5a IX'5a VII'7c IX'7c  SP3 KI3 LU8 KI7 | IXs | | V'5c IX'5c VII'7a IX'7a  SP3 KI3 LU8 KI7 | Vs | VII'7a V'7a IX'9c V'9c  LU8 SP5 KI10 SP9 | Vp | | VII'7c V'7c IX'9a V'9a  LU8 SP5 KI10 SP9 |  |
|  | Xp | | VI'6a X'6a VIII'8c X'8c  ST36 BL40 LI1 BL67 | Xs | | VI'6c X'6c VIII'8a X'8a  ST36 BL40 LI1 BL67 | VIs | VIII'8a VI'8a X'10c VI'10c  LI1 ST45 BL66 ST44 | VIp | | VIII'8c VI'8c X'10a VI'10a  LI1 ST45 BL66 ST44 |  |
|  | VIIp | | III'3a VII'3a IX'9c VII'9c  HT8 LU10 KI10 LU5 | VIIs | | III'3c VII'3c IX'9a VII'9a  HT8 LU10 KI10 LU5 | VIIs | V'5a VII'5a I'1c VII'1cSP3  LU9 LR1 LU11 | VIIp | | V'5c VII'5c I'1a VII'1a  SP3 LU9 LR1 LU11 |  |
|  | VIIIp | | IV'4a VIII'4a X'10c VIII'10c SI5 LI5 BL66 LI2 | VIIIs | | IV'4c VIII'4c X'10a VIII'10a SI5 LI5 BL66 LI2 | VIIIs | VI'6a VIII'6a II'2c VIII'2c  ST36 LI11 GB41 LI3 | VIIIp | | VI'6c VIII'6c II'2a VIII'2a  ST36 LI11 GB41 LI3 |  |
| IF* | IIIs | | V'5a III'5a VII'7c III'7c  SP3 HT7 LU8 HT4 | IIIp | | V'5c III'5c VII'7a III'7a  SP3 HT7 LU8 HT4 | Ip | VII'7a I'7a IX'9c I'9c  LU8 LR4 KI10 LR8 | Is | | VII'7c I'7c IX'9a I'9a  LU8 LR4 KI10 LR8 |  |
| B* | IVs | | VI'6a IV'6a VIII'8c IV'8c  ST36 SI8 LI1 SI1 | IVp | | VI'6c IV'6c VIII'8a IV'8a  ST36 SI8 LI1 SI1 | IIp | VIII'8a II'8a X'10c II'10c  LI1 GB44 BL66 GB43 | IIs | | VIII'8c II'8c X'10a II'10a  LI1 GB44 BL66 GB43 |  |
| IZ* | Vs | | III'3a V'3a IX'9c V'9c  HT8 SP2 KI10 SP9 | Vp | | III'3c V'3c IX'9a V'9a  HT8 SP2 KI10 SP9 | IXp | V'5a IX'5a I'1c IX'1c  SP3 KI3 LR1 KI1 | IXs | | V'5c IX'5c I'1a IX'1a  SP3 KI3 LR1 KI1 |  |
| V* | VIs | | IV'4a VI'4a X'10c VI'10c  SI5 ST41 BL66 ST44 | VIp | | IV'4c VI'4c X'10a VI'10a  SI5 ST41 BL66 ST44 | Xp | VI'6a X'6a II'2c X'2c  ST36 BL40 GB41 BL65 | Xs | | VI'6c X'6c II'2a X'2a  ST36 BL40 GB41 BL65 |  |
| P* | III’s | | III'5a III'9c  HT7 HT3 | III’p | | III'5c III'9a  HT7 HT3 | $\bar{\mathrm{III}}$’p | $\bar{\mathrm{III}}$'7a $\bar{\mathrm{III}}$'1c  PC5 PC9 | $\bar{\mathrm{III}}$’s | | $\bar{\mathrm{III}}$'7c $\bar{\mathrm{III}}$'1a  PC5 PC9 |  |
|  | IV’s | | IV'6a IV'10c  SI8 SI2 | IV’p | | IV'6c IV'10a  SI8 SI2 | $\bar{\mathrm{IV}}$’p | $\bar{\mathrm{IV}}$'8a $\bar{\mathrm{IV}}$'2c  TE1 TE3 | $\bar{\mathrm{IV}}$’s | | $\bar{\mathrm{IV}}$'8c $\bar{\mathrm{IV}}$'2a  TE1 TE3 |  |

EC: Eight Constitution

* ; In Kuon Do-won’s 1974 paper,
F* = Fundamental Formula,
V = Vitalization Formula,
B = Bactericidal Formula,
P = Psycho Formula,
IZ = Inflammation Formula on Zang system, s
IF = Inflammation Formula on Fu system.
Other formulas were not named.

****** ; 'p' = promote(補), 's' = suppress(瀉), 'a' = anadromous(迎→瀉), 'c' = catadromous(隨→補)

**D. Eight Constitution Lifestyle Intervention(1)**

# Hepatonia Regimen

When you are healthy, you sweat a lot: however, when you are weak, you do not sweat. Your body feels lighter when you sweat from various activities: that is because your constitution requires a lot of perspiration. Therefore, enjoying hot baths all the time is a good practice for your health. Mountain hiking and moderate speaking are good. Having higher blood pressure that average is a healthy state for you.

## Harmful Regimen

All kinds of ocean fishes and shellfishes, Raw cabbages and most Green and leafy Vegetables, Dextrose, Cocoa/Chocolate, Buckwheat, Bracken(fern), Persimmon, Quince, Cherry, Grapes(Wine), Swim(cold bath), Aloe vera, Dextrose injection

## Beneficial Regimen

All kinds of Meats, Rice, Soybean, Wheat, Indian millet, Root Vegetables(radish, carrot, yam, lotus root and taro), Coffee, Milk(warm), Garlic, Pumpkin or squash, Mushrooms, Sugar, Some fresh water fishes(eel, loach/mudfish), Alkaline beverages, Pear, Apple, Watermelon, Nuts(walnut, pecan, chestnut, pine-nut), Deer horn(herb), Ginseng, Vitamin A, B and D

# Cholecystonia Regimen

Discomfort in your lower abdomen is large intestinal inertia, which is the very cause of such symptoms as a heavy feeling in your legs, backache, irregular bowel movements, depression, cold body and occasional insomnia. Therefore, it is good to keep your lower abdomen warm all the time. As your constitution is prone to becoming alcoholic, it is necessary to take extra precautions in consuming alcohol.

## Harmful Regimen

All kinds of ocean fishes and shellfishes, Alcoholic beverages, Raw cabbages and most Green and Leafy Vegetables, Buckwheat, Bracken(fern), Cocoa/Chocolate, Grapes(Wine), Cherry, Persimmon, Quince, Dextrose, Ginseng, Swim(cold bath)

## Beneficial Regimen

Beef, Pork, Rice, Soybean, Wheat, Indian millet, Root Vegetables(radish, carrot, yam, lotus root and taro), Coffee, Milk(warm), Garlic, Pumpkin or squash, Mushrooms, Sugar, All kinds of nuts(walnut, pecan, chestnut, pine-nut), Some fresh water fishes(eel, loach/mudfish), Alkaline beverages, Pear, Melons, Deer horn(herb), Squalene oil, Vitamin A and D

# Pancreotonia Regimen

Your health is so directly related to your tendency to be quick-tempered that your health practice should always be to keep your poise and not to hurry. Although your constitution has strong digestive powers, you should avoid food that doesn’t suit your constitution. Alcohol and cold baths are detrimental.

## Harmful Regimen

Chicken and Poultry, Lamb, Brown rice, Seaweeds, Apple, Orange/Citrus, Mango, Ginseng, Potato, Honey, Vitamin B group, Pepper, Ginger, Green onion, Onions, Mustard, Cinnamon, Curry, Hot & Spices, Sesame oil, Dates, Digestive enzyme, Antibiotics, Cold bath

## Beneficial Regimen

Barley, Rice, Eggs, Wheat, Beans and nuts, Red beans, Pork, Beef, Green Vegetables, All kinds of fishes and shellfishes, Persimmon, Pear, Melons, Strawberry, Cranberry and most Berries, Banana, Vitamin E, Ice, Aloe vera, Mushrooms

# Gastrotonia Regimen

You have to be careful with medications and foods since this constitution is prone to indigestion due to side effects from them. Cool, fresh food is beneficial while alcohol and cold baths are detrimental.

## Harmful Regimen

Sweet/Brown rice, Chicken and Poultry, Lamb, Mustard, Red and black pepper, Cinnamon, Curry, Ginger, Green onion, Hot & Spices, Apple, Orange/Citrus, Mango, Tomato, Seaweed, Burnt foods, Ginseng, Dates, Honey, Vitamin B group, Penicillin, Alcohol, Smoking cigarette

## Beneficial Regimen

Barley, Rice, Red beans, Mung beans, Cucumber, Green Vegetables, All kinds of ocean fishes and shellfishes, Swellfish, Pork, Beef, Persimmon, Melons, Pineapple, Grapes, Strawberry, Banana, Aloe vera, Ice, Chocolate, Vitamin E

# Pulmotonia Regimen

The reason why medication is as effective as it is harmful and your body suffers after eating meat is because your liver is functioning weakly. Therefore, your healthy foods should be vegetables and seafood. The secret to your health is spending a lot of time standing with your back straight. Avoid sunbathing and excessive perspiration.

## Harmful Regimen

All kinds of Meats, Milk, All fresh water fishes, Coffee/Tea, Artificial seasonings/drinks, Wheat flour, Indian millet, Pumpkin or squash, Peppers, Garlic, Mushrooms, Sugar, Root Vegetables, Soy bean, All kinds of Nuts, Apples, Pear, Deer horn(herb), Ginseng, All medicinal substances, Vitamin A, B and D, Alkaline beverages, Alcohol and Cigarette, Hot Bath(Sauna)

## Beneficial Regimen

All kinds of ocean fishes and shellfishes, Rice(White), Buckwheat, Mung beans, Mugwort, Cucumber, Eggplant, Cabbage, Lettuce, Green Vegetables, Bracken(fern), Dextrose, Cocoa/Chocolate, Banana, Strawberry, Peach, Cherry, Persimmon, Quince, Aloe vera, Ice, Dextrose injection

# Colonotonia Regimen

The first health principle for this constitution is to stop eating any kind of meat and the second is not to use medication. The third is not to get upset. In case you have muscle inertia symptoms, extra precaution is required and it’s beneficial to take cold baths regularly.

## Harmful Regimen

All kinds of Meats, Milk, Garlic, Deer horn(herb), All fresh water fishes, Artificial seasonings, Wheat flour, Indian millet, Pumpkin or squash, Soy bean, Sugar, Pear, Apples, Melons, Chestnut, Pine-nut, Ginkgo, All kinds of Nuts, Root Vegetables, Mushrooms, Vitamin A, D and E, Alkaline beverages, Hot Bath(Sauna)

## Beneficial Regimen

Buckwheat, Rice(White), Dextrose, All kinds of ocean fishes and shellfishes(except oyster), Green Vegetables, Cucumber, Bracken(fern), Green seaweed, Grapes, Peach, Persimmon, Cherry, Pineapple, Strawberry, Mustard, Cocoa/Chocolate, Acanthopanax root bark, Swim(cold bath)

# Renotonia Regimen

The reason you are healthier in the colder seasons is because you should not sweat a lot constitutionally. Therefore, the only healthy way to avoid perspiration is to enjoy cold baths or rubbing your body with a cold towel.

## Harmful Regimen

Barley, Red beans, Cucumber, Pork, Oyster and shellfishes, Persimmon, Melons, Strawberry, Banana, Pineapple, Beer, Ice, Vitamin E, Cranberry, Aloe vera, Mushrooms, Mercury, Hot Bath(Sauna)

## Beneficial Regimen

Sweet/Brown rice, Chicken and Poultry, Lamb, Beef, Seaweeds, Cinnamon, Ginger, Green onion, Mustard, Red and black pepper, Sesame oil, Potato, Apple, Mango, Orange/Citrus, Tomato, Ginseng, Honey, Dates, Vitamin B group

# Vesicotonia Regimen

If you have food cold in temperature as well as quality, your cold stomach will become colder and lead you to unhealthy and anxious states, eventually to gastroptosis. Therefore, your first health principle should be light eating and eating warm food.

## Harmful Regimen

Barley, Red beans, Cucumber, Pork, Eggs, Swellfish, All kinds of shellfishes, Persimmon, Melons, Banana, Strawberry, Grapes(Wine), Beer, Chocolate, Aloe vera, Ice, All types of cold beverages and foods, Vitamin E, Alkaline Beverages, Mecury, Smoking cigarette, Hot bath(Sauna)

## Beneficial Regimen

Sweet/Brown rice, Potato, Corn, Sesame oil, Seaweeds, Chicken and Poultry, Lamb, Red and black pepper, Mustard, Cinnamon, Curry, Green onion, Ginger, Apple, Orange/Citrus, Tomato, Mango, Ginseng, Dates, Honey, Vitamin B group

**References**

1. Yun J. Protocol development of Eight Constitution Medicine registry for chronic low back pain [thesis]. [Sungkyunkwan University Graduate School]: Sungkyunkwan University; 2022.

2. Kim C, Hong KW, Park DH, Chun S, Oh S, Park Y, et al. Lung- and liver-dominant phenotypes of Korean eight constitution medicine have different profiles of genotype associated with each organ function. Physiological Reports. 2022;10(17):e15459.

3. Kuon D. Studies on constitution-acupuncture therapy. J Myong Ji University. 1974;7:583–606.

4. Kuon D. A Theoretical Basis for The Eight Constitution Acupuncture. Adv Med Bio. 2010;5:243–5.

5. Shin YS, Nah SS, Oh HS, Park YJ, Park YB. A study on consistency and accuracy of pulse diagnosis in Eight-Constitution Medicine. Oriental Pharmacy and Experimental Medicine. 2009;9(1):14–9.

**The content of Reference 1 was translated from the original Korean thesis into English and utilized in this supplementary file with the author’s permission.*
